# A Monster

**Published:** 1867-11

**Authors:** C. G. Simmons

**Affiliations:** Student of the Baltimore College of Dental Surgery.


### ARTICLE VI.

### *A Monster.*

LEXINGTON, C. H., S. C., *July* 8, 1867.

*Editors American Journal Dental Science.*

GENTLEMEN :

A MONSTROSITY of no ordinary interest having recently come under my notice, I have thought it might prove sufficiently worthy of record to find a place in the columns of your Journal.

On the first of the present month there was born in this village a child having the following peculiarities. Of the face the bones in general are perfect except the maxillæ which present not the slightest trace of alveolar processes, the tongue, which is rather large, resting directly upon the lip and almost protruding beyond it. There is also a longitudinal fissure of the hard palate. The orbits, large in size, protrude to a frightful extent, so that the malar and frontal bones seem continuous, and the globes of the eye resemble two hemispheres resting upon the single bone thus formed, as though a sphere had been divided in half, and the two segments laid on a plane resting each upon the flat surface of the section. Underneath the lids, however, which are never closed, no natural eye is discernable, but only a spherical sac cantaining a dirty yellowish brown pus-like substance, similar to that which it is constantly discharging in small quantities.

The temporal bones are but partially developed, but there are four parietals, perfectly formed, unusually large, and semi-oval in shape. The occipital bone appears to be single, but extremely large, rather oblong, or perhaps rectangular. The frontal is as large as the whole face below it, and is polygonal in figure.

The left eye is remarkably mobile. By crying a convulsive action is excited which protrudes the entire ball from its socket, the lids being behind it. Again it is immediately retracted to its former position, the lids partially closing over it. Respectfully,

C. G. Simmons,

Student of the Baltimore College of Dental Surgery.

---

### CORRESPONDENCE.

---

